# Influence of the ECMO circuit on the concentration of nutritional supplements

**DOI:** 10.1038/s41598-020-76299-5

**Published:** 2020-11-06

**Authors:** Beate Rikken Lindberg, Vibeke Videm, Thorleif Dahl, Gro Sørensen, Arnt Eltvedt Fiane, Amrit Singh Thiara

**Affiliations:** 1 Department of Cardiothoracic Surgery, Oslo University Hospital, Postboks 4950 Nydalen, 0424 Oslo, Norway; 2 Department of Clinical and Molecular Medicine, NTNU-Norwegian University of Science and Technology, Trondheim, Norway; 3 Department of Immunology and Transfusion Medicine, St. Olavs University Hospital, Trondheim, Norway

**Keywords:** Medical research, Biomedical engineering, Cardiac device therapy

## Abstract

Circulating compounds such as drugs and nutritional components might adhere to the oxygenator fibers and tubing during ECMO support. This study evaluated the amount of nutritional supplements adsorbed to the ECMO circuit under controlled ex vivo conditions. Six identical ECMO circuits were primed with fresh human whole blood and maintained under physiological conditions at 36 °C for 24 h. A dose of nutritional supplement calculated for a 70 kg patient was added. 150 mL volume was drawn from the priming bag for control samples and kept under similar conditions. Blood samples were obtained at predetermined time points and analyzed for concentrations of vitamins, minerals, lipids, and proteins. Data were analyzed using mixed models with robust standard errors. No significant differences were found between the ECMO circuits and the controls for any of the measured variables: cobalamin, folate, vitamin A, glucose, minerals, HDL cholesterol, LDL cholesterol, total cholesterol, triglycerides or total proteins. There was an initial decrease and then an increase in the concentration of cobalamin and folate. Vitamin A concentrations decreased in both groups over time. There was a decrease in concentration of glucose and an increased concentration of lactate dehydrogenase over time in both groups. There were no significant alterations in the concentrations of nutritional supplements in an ex vivo ECMO circuit compared to control samples. The time span of this study was limited, thus, clinical studies over a longer period of time are needed.

## Introduction

Extracorporeal membrane oxygenation (ECMO) is used alongside conventional intensive care to support critically ill patients^1–3^. It permits treatment and recovery during severe cardiac and pulmonary failure. During the COVID-19 pandemic, this is the highest possible treatment for respiratory failure. These patients are given multiple drugs that include sedatives, antibiotics, anticoagulants, and vasoactive agents, as well as nutritional supplements. The pharmacokinetics of medication administered during ECMO is affected, with differences being observed between hydrophilic and lipophilic compounds^2,3^.

Nutritional support is of great importance during ECMO given that these patients are some of the most severely ill and are most likely to have a prolonged stay on the intensive care unit. Furthermore, nutritional requirements may increase due to increased protein catabolism secondary to inflammation and acute illness^4^. There is much debate surrounding optimum nutrition support practices for critically ill patients^5,6^. Patients who receive ECMO support for weeks or have a poor premorbid nutritional status due to chronic disease often need parenteral nutrition. Parenteral nutrition is commonly given as a sterile emulsion of water, protein, lipid, carbohydrate, electrolytes, vitamins, and trace elements. These nutrient groups have different sizes, charge, and binding properties. The use of parenteral nutrition in patients receiving ECMO support remains controversial because of the possibility of lipid infiltration into the oxygenator causing oxygenator failure^7^. As such, patients on ECMO support may have different drug requirements and need a different nutritional strategy.

The ECMO circuit includes both polyvinyl chloride (PVC) tubing and oxygenator polymethylpentene (PMP) fibers which may bind a variety of circulating compounds such as drugs, and possibly nutrients, effectively reducing the bioavailability of these compounds. Any loss of vital nutrients due to adsorption to the ECMO circuits may lead to further nutritional debilitation in critical ill patients. This is important because there is growing evidence that malnutrition in critically ill patients influences morbidity and mortality^4,8^.

In our previous experience with ECMO circuits, we found a white layer deposited on the venous side of the oxygenator over time, we assumed this to consist of drugs or nutrients^9^. In the ECMO circuits we used previously, the centrifugal pump and oxygenator were separated and the venous side of the oxygenator was accessible for visual inspection during ECMO support^9^. We presently use the Cardiohelp and HLS advanced 5.0 systems with an integrated centrifugal pump and oxygenator (Maquet Cardiopulmonary GmbH, Rastatt, Germany). The HLS module has a priming volume of 240 mL and can handle flow rates from 0.5 to 5 L/min, being suitable for both pediatric and adult patients (Table 1). It is clearly a much more advanced system with integrated sensors for pressure, venous oxygen saturation and temperature^10^. However, the design makes it impossible to observe for color change on the venous side of oxygenator as a sign of deposition on the membrane. We therefore performed an ex vivo study to examine if any nutritional supplements given were adsorbed in the ECMO circuit.

**Table 1:** Description of the ECMO circuit.

Technical data	HLS set advanced 5.0
Flow rate	0.5–5 L/min
Gas exchange surface area	1.3 m^2^
Heat exchange surface area	0.3 m^2^
Prime volume (HLS module advanced)	240 mL
Coating	Bioline
Membrane material	Polymethylpentene
Tubing set	Polyvinylchloride
Priming bag	Polyvinylcholride

*HLS *Heart–lung support.

## Methods

Ethical approval was obtained by the Regional Committee for Medical and Health Research Ethics (2017/2545) Oslo University Hospital, Oslo, Norway. Informed consent was obtained from each volunteer blood donor and all experiments were performed in accordance with relevant guidelines and regulations (Trial registration number 18/04990).

### ***ECMO circuits***

Six ECMO circuits (Cardiohelp system with HLS advanced 5.0 oxygenators) were used in this ex vivo experiment. They were preferred over the Permanent Life Support (PLS system (Maquet Cardiopulmonary GmbH) which we no longer use. The system was first primed with 1 L Ringer acetate (Fresenius Kabi AB Uppsala, SW), which circulated for five minutes at 3000 rotations per minute (RPM), to ensure that it was free from air emboli. Most of the Ringer acetate volume was then replaced with fresh human blood donated by volunteers and collected in CPAD (citric acid, sodium citrate, monobasic sodium phosphate, dextrose, and adenine) (Baxter Healthcare Co, Deerfield, IL, USA) collection bags. 5000 I.E Heparin (Leo Pharma, Ballerup, Denmark) was added to the circuits to keep activated clotting time (ACT) above 200 s. The size and length of Bioline-coated tubing (Maquet Cardiopulmonary AG, Rastatt, Germany) in the ECMO circuits were shortened to reduce the priming volume^11^. The final volume of the circuit was 815 mL (780–930 mL). The circuit flow rate was kept at 2 L/min, sweep-gas and O_2_ fraction levels were used to maintain blood gases within physiologically normal levels, at a temperature of 36 °C. Arterial and venous pressure and the gradient between them, was recorded at every sampling time, as an increasing pressure gradient across the oxygenator indicates clotting and depositions in the membrane^12^. After activating Kabiven Fresenius, a nutritional supplement three-chamber bag of 2100 mL (Fresenius Kabi Norge AS, Halden, Norway), the required water-soluble vitamins (Soluvit Fresenius, Fresenius Kabi Norge AS), fat-soluble vitamins (Vitalipid Fresenius, Fresenius Kabi Norge AS), and trace elements (Addaven Fresenius, Fresenius Kabi Norge AS) were added (Table 2). The recommended dosage of Kabiven Fresenius in adults is 19–38 mL/kg/day. Compared to the blood volume of a person weighing 70 kg, the lowest circuit volume was 18%. Thus, adding 15 mL of nutritional supplement of Kabiven to the circuit, was then estimated calculated to be similar to a daily dosage of 30 mL/kg/day. In clinical practice, parenteral nutrition is given as a continuous infusion. Since the main aim of the study was to examine any adsorption to the membrane, we added it as a bolus dose to be able to determine any changes over time in the samples. The nutritional supplement was injected into the ex vivo circuit two minutes after the pump was started.

**Table 2:** Nutritional supplements (Kabiven Fresenius) used in this study.

Nutrient supplements	Quantity
Volume (mL)	2025
Glucose (g)	171
Aminoacid (g)	133
Nitrogen (g)	21.2
Fat (Smoflipid) (g)	58.4
Sodium (mmol)	82.6
Potassium (mmol)	61.9
Magnesium (mmol)	10.3
Calcium (mmol)	5.2
Phosphate (mmol)	25.8
Zinc (mmol)	0.08
Sulphate (mmol)	10.4
Chloride (mmol)	72.2
Acetate (mmol)	253
Addaven^a^ (mL)	20
Soluvit^b^ (vial)	1
Vitalipid^c^ (mL)	10

Kabiven Fresenius (Fresenius Kabi Norge AS, Halden, Norway).

^a^Addaven = trace elements.

^b^Soluvit = water-soluble vitamins.

^c^Vitalipid = fat-soluble vitamins.

### Control sample

A 150 mL control sample was drawn from the priming bag of the ECMO circuit after the calculated amount of nutritional supplement had been added and circulated for one minute. Control samples were kept in a glass Erlenmeyer flask (Greiner bio one, Medi-kjemi AS, Asker, Norway) with a cotton plug on top, to prevent evaporation and anaerobe conditions. It was agitated continuously on a magnetic mixer/heater to ensure even distribution of the nutrients and kept at a temperature of 36 °C.

### Blood samples and analyses

All samples were collected from a three-way stopcock placed in the tubing directly after the oxygenator. A baseline sample was drawn five minutes after nutritional supplement infusion. To maintain blood gases within physiologically normal levels, the circuits and control flasks were kept at a temperature of 36 °C during the study period. Samples were collected simultaneously from the circuit (oxygenator group) and the control flask (control group) after 1 h, 6 h, and 24 h. Samples were drawn into sterile Vacutainer glass tubes, containing ethylenediaminetetraacetic acid (EDTA) (Greiner bio one, Medi-kjemi AS,) citrate sodium, or CTAD (citrate, theophylline, adenosine, and dipyridamole), (Greiner bio one, Medi-kjemi AS) according to the substances to be analyzed. Samples were immediately transported to the laboratory (Oslo university hospital, Oslo, Norway) for centrifugation and analysis. A total of 18 nutritional substances were analyzed over the 24-h period, including vitamins, minerals, lipids, total protein, lactate dehydrogenase (LDH) and glucose (Table 3). Blood gas analyses were performed on samples collected in 2 mL purposed syringes, using an ABL90flex analyzer (Radiometer Medical Aps, DK).

**Table 3:** Assay method for nutritional supplements.

Nutrient	Methodology	Instruments
Cobalamin (pmol/L)	Electrochemiluminescence	Roche Cobas 8000 analyzer^c^
Folate (nmol/L)	Electrochemiluminescence	Roche Cobas 8000 analyzer^c^
Vitamin A (μmol/L	Performance liquid chromatography	Dionex ultimate 3000^d^
Potassium (mmol/L)	Indirect potentiometry	Roche Cobas 8000 analyzer^c^
Calcium (mmol/L)	Colorimetry	Roche Cobas 8000 analyzer^c^
Phosphate (mmol/L)	Photometry	Roche Cobas 8000 analyzer^c^
Magnesium (mmol/L)	Colorimetry	Roche Cobas 8000 analyzer^c^
Total cholesterol (mmol/L)	Enzymatic colometry	Roche Cobas 8000 analyzer^c^
HDL^a^ cholesterol (mmol/L)	Enzymatic colometry	Roche Cobas 8000 analyzer^c^
LDL^b^ cholesterol (mmol/L)	Enzymatic colometry	Roche Cobas 8000 analyzer^c^
Triglycerides (mmol/L)	Photometry	Roche Cobas 8000 analyzer^c^
Apolipoprotein A1(g/L)	Immunoturbodimetry	Roche Cobas 8000 analyzer^c^
Apolipoprotein B(g/L)	Immunoturbodimetry	Roche Cobas 8000 analyzer^c^
Lipoprotein A (g/L)	Immunoturbodimetry	Roche Cobas 8000 analyzer^c^
Albumin (g/L)	Colorimetry	Roche Cobas 8000 analyzer^c^
Total protein (g/L)	Photometry	Roche Cobas 8000 analyzer^c^
Lactate dehydrogenase (μ/L)	Photometry	Roche Cobas 8000 analyzer^c^
Glucose (mmol/L)	Photometry	Roche Cobas 8000 analyzer^c^

^a^*HDL *High-density lipoprotein.

^b^*LDL *Low-density lipoprotein.

^c^(Roche Diagnostics, IN, USA).

^d^(Thermo Fisher Scientific Inc., MA, USA).

### Statistics

Statistical analyses were performed using mixed models with robust standard errors, which allows for repeated samples within each setup and incomplete data. Model fit was evaluated using residual plots. Data are presented as mean with 95% nonparametric confidence intervals and standard deviation. P-values < 0.05 were considered to be statistically significant.

## Results

There were no significant differences in blood gas analyses, pH, temperature, and electrolyte composition between the control and oxygenator groups. The measured pressures in the arterial and venous lines were stable.

**Figure 1:**
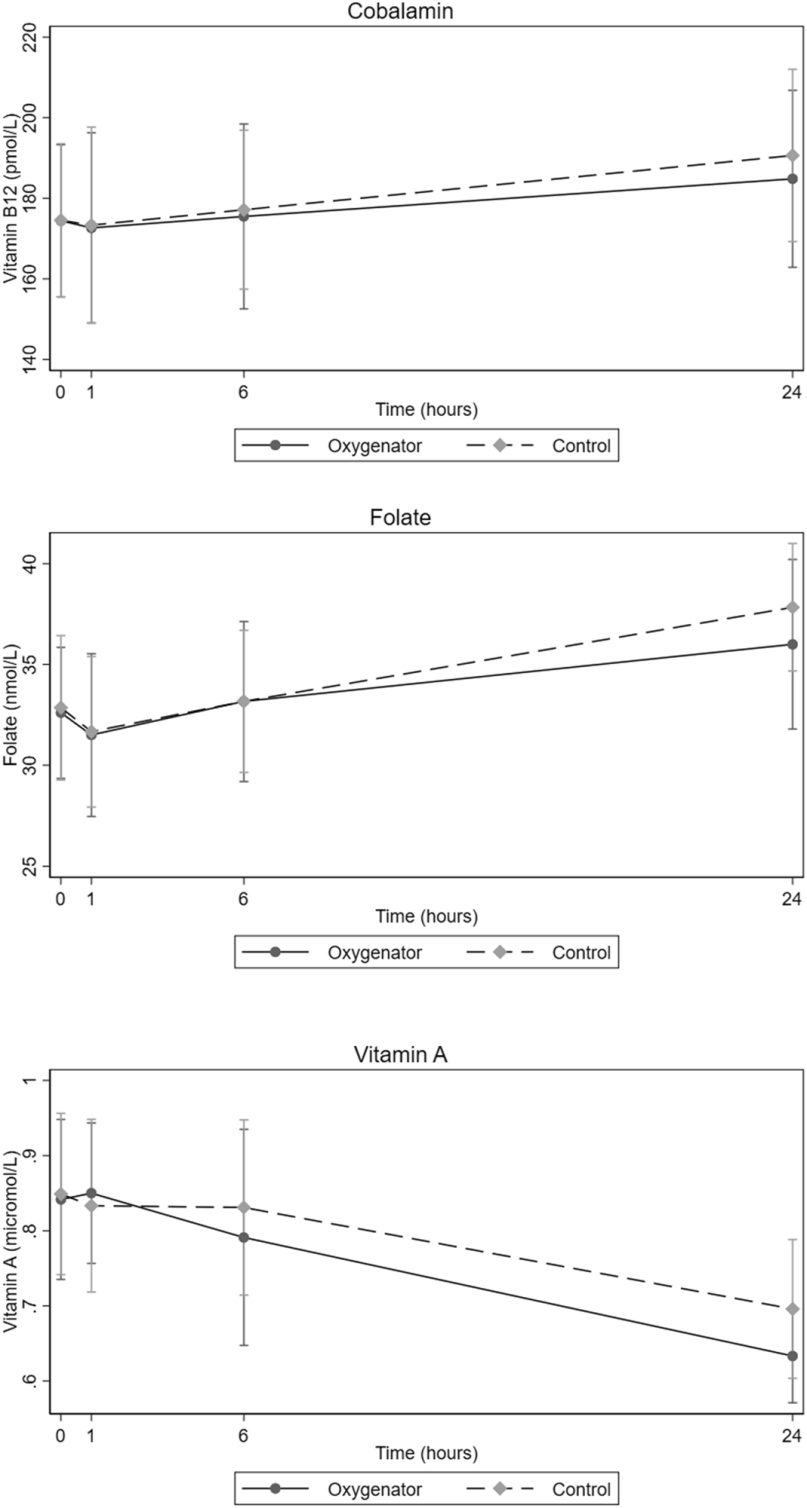
Concentration of cobalamin, folate and vitamin A at four pre-determined time points with oxygenator and control group circuits during study periods (mean with 95% confidence intervals). Blood samples were obtained at baseline (0 h), 1 h, 6 h and 24 h.

Both cobalamin and folate concentrations decreased in the first hour samples, but increased gradually over time in both groups, whereas vitamin A gradually decreased over time from baseline (Fig. 1). There were no significant differences in vitamin concentrations between the two groups (cobalamin *p* = 0.99, folate *p* = 0.92, vitamin A *p* = 0.93).

**Figure 2:**
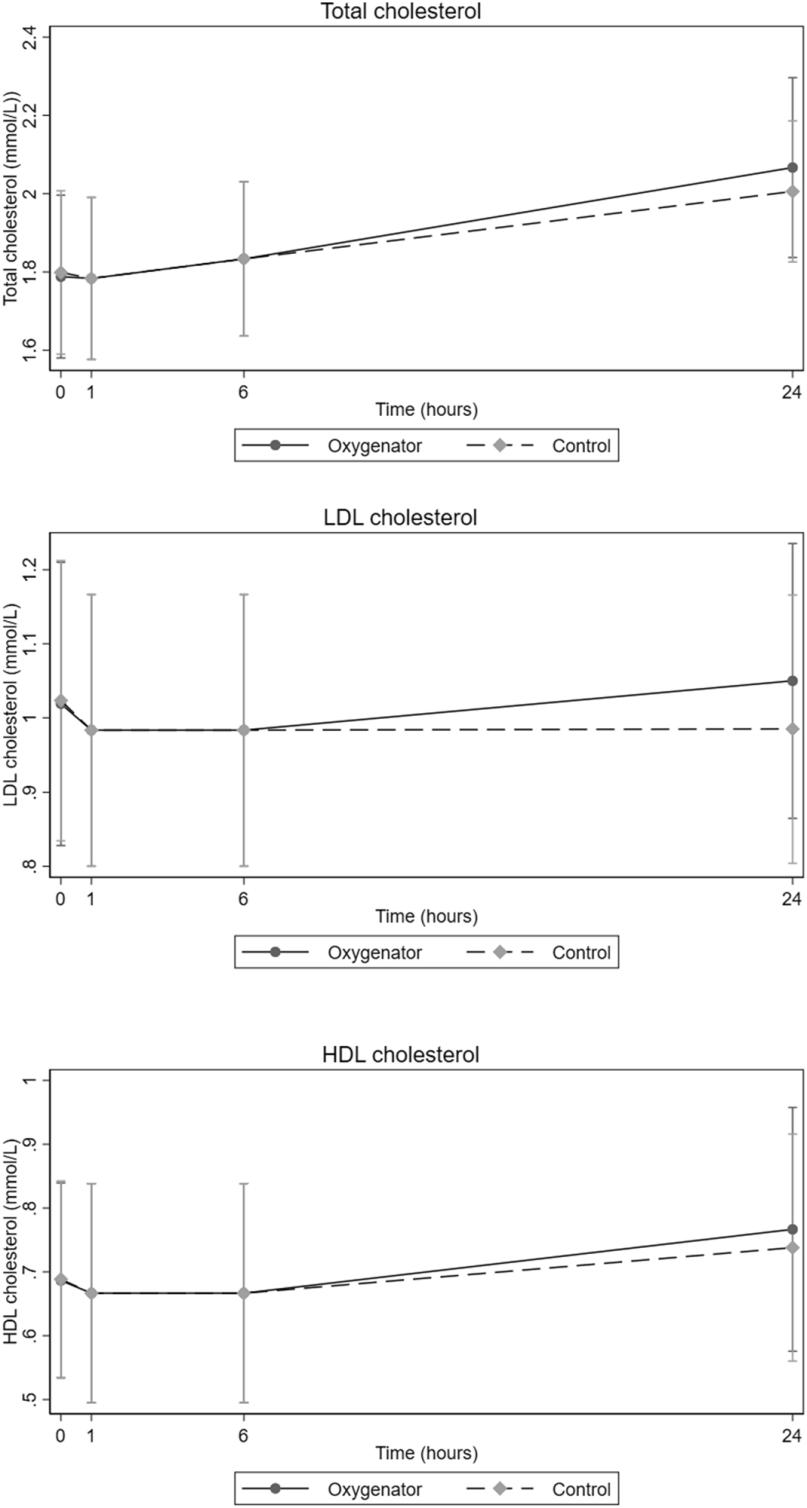
Concentration of total cholesterol, LDL cholesterol, and HDL cholesterol at four pre-determined time points with oxygenator and control group circuits during study periods (mean with 95% non-parametric confidence intervals). Blood samples were obtained at baseline (0 h), 1 h, 6 h and 24 h.

**Figure 3:**
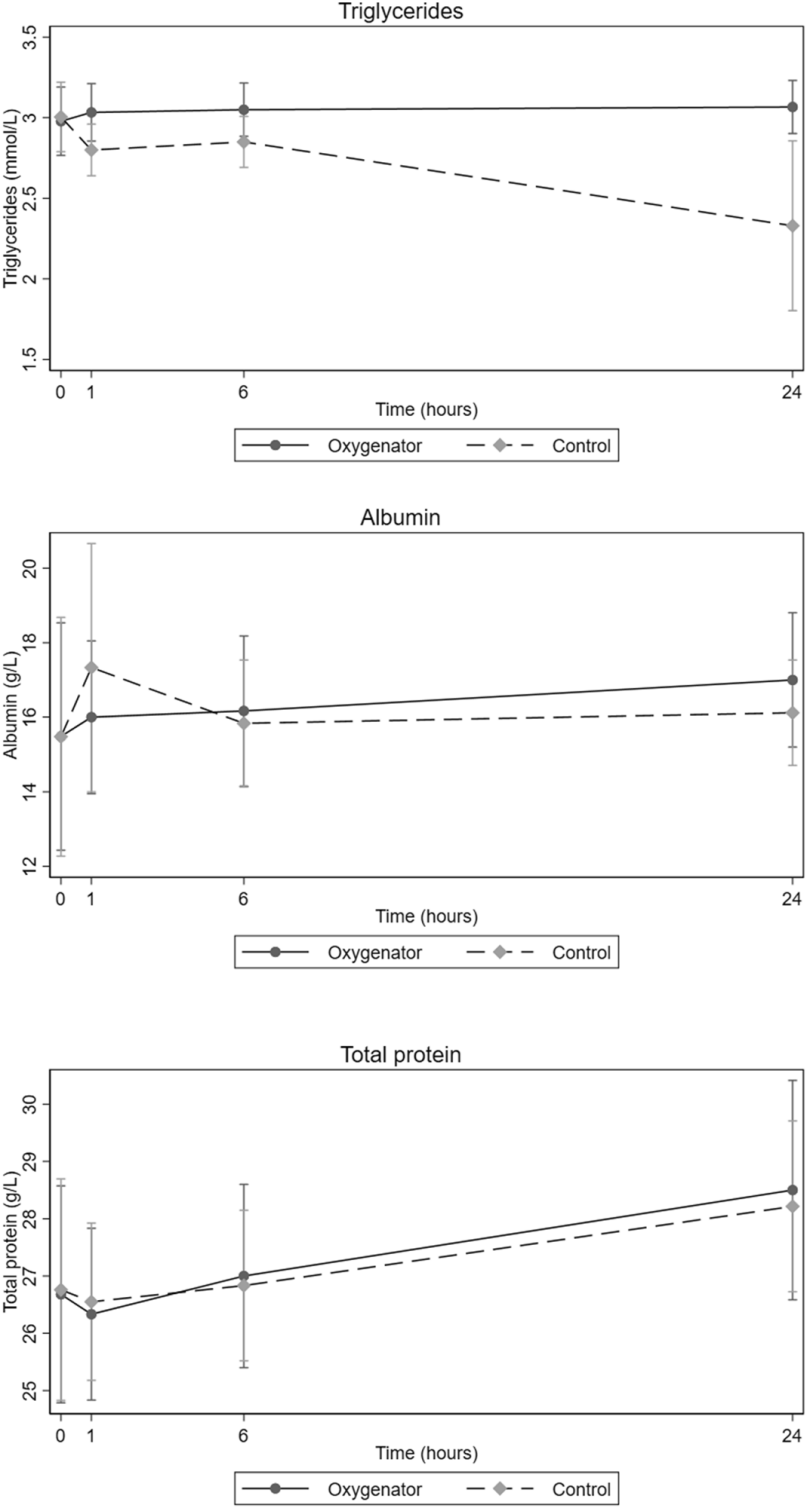
Concentration of triglycerides, albumin, and total protein at four pre-determined time points with oxygenator and control group circuits during study periods (mean with 95% non-parametric confidence intervals). Blood samples were obtained at baseline (0 h), 1 h, 6 h and 24 h.

The concentration of potassium, calcium, phosphorus, and magnesium increased in both groups in the samples taken after 24 h (Table 4). The concentrations of both calcium and magnesium decreased from baseline to one hour, and then gradually increased over time in both groups. The concentration of potassium increased from baseline until 24 h. There were no significant differences in concentrations of the minerals analyzed between the groups (potassium *p* = 0.89, calcium *p* = 0.84, phosphorus *p* = 0.63, magnesium *p* = 0.82). There were no significant differences between the groups for HDL cholesterol (*p* = 0.99), LDL cholesterol (*p* = 0.98), or total cholesterol (*p* = 0.94) (Fig. 2). Triglyceride concentrations were unchanged in the oxygenator group and decreased slightly in the control group, but no significant differences were observed between the groups (*p* = 0.86) (Fig. 3). Total protein increased over time for both groups, but with no significant difference between the groups (*p* = 0.95). LDH increased in both groups but with no significant difference between them (*p* = 1.0). The concentration of glucose decreased but without any significant difference between the circuit and the control group (*p* = 0.95).

**Table 4:** Concentrations of nutritional supplements in oxygenator and control groups.

Variable	Group^a^	Baseline		1 h		6 h		24 h		p-value
		Mean	SD^b^	Mean	SD^b^	Mean	SD^b^	Mean	SD^b^	
Cobalamin (pmol/L)	O	175.4	27.9	172.6	30.92	175.5	30.00	184.8	28.78	0.99
	C	175.8	27.9	173.3	31.89	177.1	25.80	189.2	27.40	
Folate (nmol/L)	O	31.6	3.6	31.50	5.28	33.1	5.19	36.00	5.51	0.92
	C	31.6	3.6	31.6	4.80	33.1	4.60	41.00	5.00	
Vitamin A (μmol/L)	O	0.84	0.15	0.85	0.12	0.78	0.19	0.63	0.25	0.93
	C	0.84	0.15	0.83	0.15	0.82	0.16	0.73	0.05	
Potassium (mmol/L)	O	4.34	0.11	4.46	0.19	4.50	0.18	5.21	0.27	0.89
	C	4.33	0.22	4.51	0.39	4.51	1.21	5.60	1.21	
Calcuim (mmol/L)	O	1.96	0.02	1.94	0.02	1.96	0.03	2.09	0.06	0.84
	C	1.96	0.02	1.96	0.02	1.96	0.03	2.01	0.05	
Phosphorus (mmol/L)	O	1.76	0.05	1.70	0.06	1.53	0.33	3.71	0.38	0.63
	C	1.76	0.05	1.85	0.08	1.98	0.29	3.20	0.21	
Magnesium (mmol/L)	O	0.98	0.02	0.96	0.02	0.97	0.01	1.06	0.06	0.82
	C	0.98	0.02	0.96	0.01	0.97	0.03	1.01	0.05	
Glucose (mmol/L)	O	23.94	1.19	22.71	0.89	21.05	0.65	17.80	0.72	0.95
	C	23.94	1.19	22.86	0.83	21.40	0.89	14.62	6.04	
LDH^c^ (U/L)	O	74.80	10.32	80.66	9.75	84.5	8.31	132.6	23.40	1.0
	C	74.80	10.32	74.16	8.51	99.8	11.72	250.2	93.9	
Apo a^d^ (g/L)	O	0.54	0.13	0.55	0.12	0.53	0.12	0.56	0.13	0.17
	C	0.54	0.13	0.55	0.12	0.55	0.12	0.55	0.10	
Apo b^e^ (g/L)	O	0.35	0.05	0.32	0.04	0.30	0.07	0.31	0.07	0.25
	C	0.35	0.05	0.32	0.04	0.30	0.07	0.33	0.05	
Lipo a^f^ (g/L)	O	35.25	40.21	34.00	39.02	34.5	40.61	37.00	42.9	1.0
	C	35.25	40.21	36.75	40.35	35.00	40.32	44.00	48.07	
Albumin (g/L)	O	15.80	3.9	16.00	2.60	16.10	2.60	17.00	2.36	1.0
	C	15.80	3.9	17.30	4.30	15.80	2.20	15.70	0.90	
Total protein (g/L)	O	26.80	2.6	26.30	1.90	27.0	2.0	28.50	2.50	0.95
	C	26.80	2.6	26.80	1.90	26.80	1.72	27.75	0.9	
Triglycerides (mmol/L)	O	3.00	0.25	3.03	0.23	3.05	0.21	3.06	0.21	0.86
	C	3.00	0.25	2.80	0.20	2.85	0.20	2.35	0.60	
LDL^g^ (mmol/l)	O	1.06	0.6	0.98	0.2	0.98	0.2	1.05	0.2	0.98
	C	1.04	0.2	0.98	0.2	0.98	0.2	0.97	0.29	
HDL^h^ (mmol/L)	O	0.68	0.22	0.66	0.22	0.66	0.22	0.76	0.25	0.99
	C	0.68	0.22	0.66	0.22	0.66	0.22	0.75	0.20	
Total cholesterol (mmol/L)	O	1.80	0.3	1.78	0.27	1.83	0.25	2.0	0.3	0.94
	C	1.80	0.3	1.78	0.27	1.83	0.25	2.0	0.21	

Values are mean and standard deviation. Statistical analyses were performed using mixed models with robust standard errors.

^a^*Group O *Oxygenator group. *C *Control group.

^b^*SD *Standard deviation.

^c^*LDH* Lactate dehydrogenase.

^d^*Apo a* Apolipoprotein A1.

^e^*Apo b* Apolipoprotein B.

^f^*Lipo a* Lipoprotein A.

^g^*LDL* Low-density Lipoprotein.

^h^*HDL* High-density Lipoprotein.

## Discussion

A number of studies have shown significant alterations in the concentrations of different medications in ECMO circuits with silicone or PVC tubing and a microporous oxygenator membrane^13–17^. The centrifugal pump, membrane oxygenator, and PVC tubing comprise a large surface area for potential drug and nutrient adsorption, which may lead to drug and nutrient loss over time^14–17^. The patients on ECMO support are critically ill and they need a sufficient and balanced diet to improve likelihood of a better outcome. ECMO circuits have improved over the years, for example, by the replacement of components with heparin coated PVC tubing and using a PMP diffusion membrane in the oxygenator. The composition and materials used in the ECMO circuits may play an important role in the binding characteristics of drugs and nutrients^18–22^.

In this study the concentration of many substances, including folate and cobalamin, increased over time in both groups (Fig. 1). This may be explained by evaporation (at 36 °C) from the oxygenator membrane, the priming bag in the circuit, or through the cotton plug in the glass flask. Some studies also defined insensible water loss from the oxygenator by evaporation. This phenomenon of water loss through a membrane has been theorized to be governed by a combination of diffusion and convection^23–25^.

There is a possibility that the hydrophilic nature of oxygenator membranes can adsorb hydrophilic vitamins like folate and cobalamin during ECMO support. The hydrophilic vitamins play essential roles in normal biological functions as coenzymes and antioxidants^26^. However, our study did not indicate a great deal of adsorption of these compounds to the ECMO surface. Furthermore, most adults have liver stores of cobalamin that will last for some time before deficiency develops.

The exposure of both the oxygenator group and control group to light may explain the decrease in the concentration of vitamin A across time in both groups (Fig. 1); this effect was also shown by Estensen et al.^8^. Vitamin A is known to be very sensitive to light and is degraded by photolysis, even in fat emulsion and light protected bags^27^. We also expected that the lipophilicity of vitamin A would affect its circuit disposition, but we could not find any difference in concentration of vitamin A between the control and oxygenator groups over time.

Glucose is the major energy source for red blood cells and metabolism in the erythrocytes may explain the decrease in glucose concentration and the increase in LDH concentration in both groups. Increased LDH concentration is also directly related to hemolysis in both the oxygenator and control groups, which could also explain the increase of potassium and phosphorus^28^. There was no significant difference between groups in the concentration of any of the analyzed minerals. The observed trend for minerals throughout the experiment could be explained by cellular metabolism and evaporation through the oxygenator membrane and control samples^23–25^.

The small decrease in concentration of LDL and HDL in the samples drawn after 1 h compared to the baseline samples in both groups is difficult to explain (Fig. 2). Oxidation may play some role in the decreasing concentration of LDL and HDL, but routine blood tests are unable to measure oxidized products of lipid and proteins. The concentration of total cholesterol, LDL cholesterol, and HDL cholesterol increased after 6 h in both groups, and this could be explained by the evaporation process (Fig. 2).

Shekar et al.^29^ reported that protein bound drugs appear to be significantly sequestered in an ex vivo ECMO circuit. However, we could not observe any significant differences in total protein concentration between the groups (Fig. 3). Bioline coating is a combination of heparin and albumin, which make the membrane surface more homogenous and hydrophilic thus preventing cell and protein adhesion. It has been shown that a hydrophilic surface has less protein adsorption than a hydrophobic surface^30^. In order to adsorb protein on a hydrophilic surface, the hydration sheath of the surface and protein must be penetrated. Hydrophilic surfaces are more blood-biocompatible on the basis of minimal interaction with blood components^31,32^. Our present ECMO circuit is more compact, the oxygenator and centrifugal pump are incorporated in the ECMO console, and it is not possible to observe the venous side of the oxygenator, therefore we are totally dependent on the pressure gradient over the oxygenator to evaluate whether there is any deposition. Again, this only indicates how large an area in the oxygenator is clogged and does not provide any information on what kind of deposits there are. The study was designed to assess the influence of an ECMO circuit on nutritional concentration for a limited period of time to evaluate the possibility that nutritional supplements could be adsorbed by the circuit. Since no patient is connected to the circuit, any change in nutrient metabolism or concentration over time is due to the circuit adsorption. The time period of this study is very limited in comparison to the clinical scenario where a continuous infusion of nutrients may last for days or weeks. Another factor missing in this study is the interaction of nutrients and their metabolism inside the patient. This study was only designed to measure the change in concentration of nutrients over time, so any functional and structural changes in nutritional supplements which lead to diminished effects through the ECMO circuit are beyond the scope of our study. Further multiple dose studies are needed to find the saturation point for nutritional components in the ECMO membrane.

In our study, we tried to mimic the clinical situation in a controlled atmosphere by using fresh human blood which allowed us to study the interaction of nutritional supplements with ECMO circuits. In our single dose ex vivo setup we could not find any differences in the concentration of nutritional compounds between the oxygenator group and the control group. This indicates that it is safe to use parenteral nutrition in standard dosage for critically ill patients who need cardiopulmonary support.

## Conclusion

There were no changes in the concentrations of nutritional supplements in an ex vivo ECMO circuit compared to control samples, indicating that parenteral nutrition is not adsorbed in the ECMO membrane or tubing. However, the time span of this study was limited, and the design made it impossible to investigate any functional and structural changes over time. Clinical studies over a longer period of time are needed to evaluate any detrimental effects of an ECMO circuit on nutritional supplements.

## Abbreviations

ECMO: Extracorporeal membrane oxygenation
PVC: Polyvinyl chloride
PMP: Polymethylpentene
RPM: Rotations per minute
CPAD: Citric acid, sodium citrate, monobasic sodium phosphate, dextrose, and adenine
ACT: Activated clotting time
EDTA: Ethylenediaminetetraacetic acid
CTAD: Citrate, theophylline, adenosine, and dipyridamole
LDH: Lactate dehydrogenase
HDL cholesterol: High density lipoprotein cholesterol
LDL cholesterol: Low density lipoprotein cholesterol
